# PyMS: a Python toolkit for processing of gas chromatography-mass spectrometry (GC-MS) data. Application and comparative study of selected tools

**DOI:** 10.1186/1471-2105-13-115

**Published:** 2012-05-30

**Authors:** Sean O'Callaghan, David P De Souza, Andrew Isaac, Qiao Wang, Luke Hodkinson, Moshe Olshansky, Tim Erwin, Bill Appelbe, Dedreia L Tull, Ute Roessner, Antony Bacic, Malcolm J McConville, Vladimir A Likić

**Affiliations:** 1 Bio21 Molecular Science and Biotechnology Institute, The University of Melbourne, Parkville, Victoria, 3010, Australia; 2 Metabolomics Australia, Bio21 Institute, The University of Melbourne, Parkville, Victoria, 3010, Australia; 3 Victorian Life Science Computational Initiative, The University of Melbourne, Parkville, Victoria, 3010, Australia; 4 National ICT Australia (NICTA), The University of Melbourne, Parkville, Victoria, 3010, Australia; 5 Centre for Astrophysics & Supercomputing, Swinburne University of Technology, Hawthorn, Victoria, 3122, Australia; 6 Walter and Eliza Hall Institute of Medical Research, 1 G Royal Parade, Parkville, Victoria, 3052, Australia; 7 Australian Centre for Plant and Functional Genomics, School of Botany, The University of Melbourne, Parkville, Victoria, 3010, Australia; 8 Victorian Partnership for Advanced Computing, 110 Victoria Street, Carlton South, Victoria, 3053, Australia; 9 ARC Centre of Excellence in Plant Cell Walls, School of Botany, The University of Melbourne, Parkville, Victoria, 3010, Australia; 10 Department of Biochemistry and Molecular Biology, The University of Melbourne, Parkville, Victoria, 3010, Australia

## Abstract

**Background:**

Gas chromatography–mass spectrometry (GC-MS) is a technique frequently used in targeted and non-targeted measurements of metabolites. Most existing software tools for processing of raw instrument GC-MS data tightly integrate data processing methods with graphical user interface facilitating interactive data processing. While interactive processing remains critically important in GC-MS applications, high-throughput studies increasingly dictate the need for command line tools, suitable for scripting of high-throughput, customized processing pipelines.

**Results:**

PyMS comprises a library of functions for processing of instrument GC-MS data developed in Python. PyMS currently provides a complete set of GC-MS processing functions, including reading of standard data formats (ANDI- MS/NetCDF and JCAMP-DX), noise smoothing, baseline correction, peak detection, peak deconvolution, peak integration, and peak alignment by dynamic programming. A novel common ion single quantitation algorithm allows automated, accurate quantitation of GC-MS electron impact (EI) fragmentation spectra when a large number of experiments are being analyzed. PyMS implements parallel processing for by-row and by-column data processing tasks based on Message Passing Interface (MPI), allowing processing to scale on multiple CPUs in distributed computing environments. A set of specifically designed experiments was performed in-house and used to comparatively evaluate the performance of PyMS and three widely used software packages for GC-MS data processing (AMDIS, AnalyzerPro, and XCMS).

**Conclusions:**

PyMS is a novel software package for the processing of raw GC-MS data, particularly suitable for scripting of customized processing pipelines and for data processing in batch mode. PyMS provides limited graphical capabilities and can be used both for routine data processing and interactive/exploratory data analysis. In real-life GC-MS data processing scenarios PyMS performs as well or better than leading software packages. We demonstrate data processing scenarios simple to implement in PyMS, yet difficult to achieve with many conventional GC-MS data processing software. Automated sample processing and quantitation with PyMS can provide substantial time savings compared to more traditional interactive software systems that tightly integrate data processing with the graphical user interface.

## Background

Gas chromatography (GC) coupled with mass spectrometry (MS) is frequently used in metabolomics [1-5]. GC-MS is best suited for the analysis of compounds of low-to-medium polarity, and can directly analyze naturally occurring volatile metabolites, as well as semi-volatile and non-volatile metabolites after derivatization [2-5]; the most widely used derivatization methods being either trimethylsilylation or *tert*-butyldimethylsilylation with oximation of keto-groups [3,6]. The type of ionization most often used in GC-MS is electron impact (EI). This type of ionization produces unstable charged molecules that undergo complex cascades of fragmentation; the m/z ratios of resulting charged fragments give the observed mass spectra. The EI mass spectra at standard 70 eV employed by most GC-MS instruments are moderately reproducible, facilitating library searches for analyte identification [3]. GC-MS has a long history in metabolic profiling of biological material [7], and currently is widely used in biochemical [1,4,8], agricultural [9], environmental [10], and biomedical research [11], as well as in a variety of industrial applications [12,13].

The principles for processing of MS-based metabolomic data are well established [3,14]. GC-MS data is acquired as a time series of mass spectral scans, where each scan consists of a series of (m/z, intensity) pairs. Typically, the raw data is transformed into a two-dimensional matrix by binning raw scans at equidistant m/z intervals. The resulting data intensity matrix is populated with binned m/z intensities, where its indices represent scan numbers (or time points) and m/z values. Subsequent processing steps may involve any combination of noise smoothing, baseline correction, peak detection, alignment, normalization, library matching, and optionally visualization. A variety of software tools have been developed for the processing of GC-MS data. These tools can generally be divided into two categories: (*a*) commercial software, either provided by the manufacturers of MS equipment or by independent vendors; (*b*) freely available software packages developed by academic groups. Examples of the commercial MS software include MassLynx (Waters Corporation, Milford, MA), ChromaTOF (Leco Corporation, St. Joseph, MI), ChemStation (Agilent Technologies, Santa Clara, CA), AnalyzerPro (SpectralWorks, Runcorn, United Kingdom), ClearView (Markes International, Rhondda Cynon Taff, UK), and IonSignature (Ion Signature Technology, N. Smithfield, RI). Typically, this type of software is mature, puts emphasis on the graphical user interface (GUI) and the overall ease of use. Many commercial MS software packages come bundled with MS hardware, thus ensuring their wide exposure to the research community. In recent years a number of freely available software packages for GC-MS data processing has been developed by the academic community. This type of software is increasingly provided under open source license [15-19].

In recent years, several MS processing/analysis software platforms that operate through the Web interface were proposed. This type of software includes SpectConnect [20], MetaboAnalyst [21,22], XCMS online [15], and MetabolomeExpress [23]. SpectConnect aims for finding of conserved components and biomarkers [20], and on the input takes putative components pre-extracted by AMDIS [24]. The XCMS online project provides access to pre-processing of LC-MS data by XCMS [15] through Web-interface. MetaboAnalyst provides the entire pipeline for high-throughput metabolomics studies [21,22], from raw data processing to statistical analyses, and for data pre-processing relies on XCMS [15]. Web-based software offers an immediate access to the processing functions with the availability of Internet, this being its greatest advantage. For such software the available computing power (typically provided by the host institution) can be a bottleneck for large processing tasks. Furthermore, if many simultaneous users are requesting the service the available computing power may be exhausted, as well as data transfer bandwidth if large data sets are required to be uploaded for processing.

AMDIS is one of the oldest, freely available stand-alone software package for the processing of GC-MS data [24]. Recently several extensions to AMDIS were proposed to improve its mode of operation [25,26]. MSFACTs [27], its successor MET-IDEA [28], as well as TagFinder [29] are specialist tools for GC-MS data analysis that combine elements of traditional pre-processing with data analysis, and in some cases spectral matching for component identification. The software packages MZmine [16], MetaQuant [17], MetAlign [30], MetaboliteDetector [18], and OpenChrom [19] incorporate both advanced algorithms and a graphical user interface (GUI), therefore being particularly suitable for interactive processing work. MZmine [16], MetaQuant [17], Maltcms/ChromA [31], and OpenChrom [19] were developed in Java, ensuring a high level of cross-platform compatibility. MetaboliteDetector was developed in C++, and relies on the Qt development framework to ensure cross-platform compatibility [18]. XCMS [15], TargetSearch [32], and centWave [33] operate within the R statistical environment (R-project, http://www.r-project.org/). MeDDL is a software prototype developed within the Matlab environment (The MathWorks, Natick, MA), with the idea to be subsequently translated into the general purpose, object oriented scripting language Python [34]. mMass is a recent software package also developed in Python, with the focus on LC-MS data [35].

Most software for GC-MS data processing is stand-alone, providing a tight integration of processing methods with GUI-based components, best suited for interactive data processing [16-19,24,30]. Alternatively, some prominent software packages consist of a collection of scripts integrated with well established computing environments, such are R or Matlab [15,32-34]. Yet an alternative approach was taken by Maltcms/ChromA: Maltcms provides a Java-based data processing engine, and ChromA is an implementation of a GUI-based interface that allows easy access to the Maltcms data processing capabilities [31]. This approach offers advantages when high-throughput, custom processing pipelines are required because the data processing engine is decoupled from GUI components and can be easily scripted.

Here we describe PyMS, a novel GC-MS processing software that decouples data processing methods from GUI-based interface. PyMS currently implements a complete set of methods required in typical GC-MS data processing, including reading of standard data formats, baseline correction, nose filtering, peak detection, peak deconvolution, peak integration, and peak alignment based on dynamic programming described previously [36]. We present details of the PyMS implementation, give an overview of current data processing capabilities, and analyze real-life data analysis scenarios based on custom data sets. For the purpose of PyMS evaluation we have designed several experiments, including a mixture of 45 metabolites representing a variety of chemical classes (sugars, organic acids, amino acids, sugar phosphates), and a series of experiments where the sample was foetal calf serum spiked with quantitative amounts of metabolite standards. The performance of PyMS was compared to several leading software packages, including AMDIS [24], one of the most widely used freely available software for GC-MS data processing [37-39]; XCMS [15], representing the new generation software for MS data processing implemented in R; and AnalyzerPro (SpectralWorks, Runcorn, United Kingdom) a widely used commercial GC-MS software package. We show that when considered in realistic data processing scenarios PyMS performance is robust, and compares favorably to these software packages.

## Implementation

### Data processing capabilities

#### Instrument data input

PyMS supports two standard data formats for data input, ANDI-MS [40] and JCAMP-DX [41]. JCAMP-DX format for chromatography and MS was developed by the IUPAC Electronic Data Standards sub-committee [41]. The ANDI-MS format [40], developed by the Analytical Instrument Association (AIA) and the American Society for Testing and Materials (ASTM), is currently one of the most widely used vendor-neutral formats in GC-MS [6]. ANDI-MS is a binary format which relies on the Network Common Data Form (NetCDF) specification [42] while JCAMP-DX is a flat-file ASCII format [41] (therefore ANDI-MS is significantly more compact). Most vendors of MS instrumentation provide software that supports export to one or both of these formats, and third party software packages can convert data stored in proprietary formats to ANDI-MS or JCAMP-DX (e.g., FileTranslatorPro, ChemSW, Fairfield, CA).

#### Raw data object

Most GC-MS data processing software performs automated binning of raw data on-the-fly, while reading the raw data file. This prevents a user from accessing raw mass spectral scans at all. However, the ability to access and manipulate raw GC-MS data may be important in many advanced data processing scenarios. Reading the raw data in PyMS results in an object designed for the optimal representation of raw instrument data named "GCMS_data" (Listing 1). PyMS internal data structures have been optimized to wrap tightly around the natural structure of raw instrument data, and to provide a significant flexibility for downstream processing (including binning).

>> > from pyms.GCMS.IO.ANDI.Function import ANDI_reader

>> > raw_data = ANDI_reader(“gc01_0812_066.cdf”)

- > Reading netCDF file ‘gc01_0812_066.cdf’

**Listing 1.** An interactive reading of a raw data file from the command line of the Python interpreter. The resulting variable named "raw_data" is a "GCMS_data" object, and holds the raw data as a list of mass spectral scans, thus closely supporting the native structure of raw data.

The "GCMS_data" object exposes key attributes of raw data, and has several special methods for accessing and manipulating the data. For example, the user can retrieve an individual raw mass spectral scan either by index or by retention time, and then retrieve the list of m/z values or measured intensities associated with this scan. In addition, the user can retrieve the entire vector of retention times, calculate the TIC (Total Ion Chromatogram), or request a compact data description to be printed out (for example see Listing 2).

>> > raw_data.info()

Data retention time range: 5.093 min -- 66.795 min

Time step: 0.375 s (std = 0.000 s)

Number of scans: 9865

Minimum m/z measured: 50.000

Maximum m/z measured: 599.900

Mean number of m/z values per scan: 56

Median number of m/z values per scan: 40

**Listing 2.** The example output when the "info()" method of the raw data object is invoked. It is assumed that that object "raw_data" was created as shown in the Listing 1.

The "trim()" method allows one to trim the data between scans, specified either as scan numbers or as retention times (see Listing 3). These are examples of methods not readily available in standard GC-MS processing software.

>> > raw_data.trim(1000, 2000) # trim between scans 1000 and 2000

>> > raw_data.trim(“6.5m”, “21m”) # trim between 6.4 and 21 minutes

**Listing 3.** Example of trimming the raw data. Trimming can be achieved either by scan number or by specifying the retention time in seconds or minutes. In this example, the first command will trim the raw data between scans 1000 and 2000. The second command will trim the raw data between 6.4 min and 21.0 min. The result of these operations is again a "GCMS_data" object, with all mass spectral scans outside the specified range removed. It is assumed that that object "raw_data" was created as shown in the Listing 1.

Once GC-MS raw data is read by the processing software, it is rarely necessary to export the raw data again; rather, the ability to output binned, processed, and extracted data becomes critical. PyMS supports export of raw data to CSV format, while binned and processed data can be exported to several vendor neutral formats (see below).

#### Raw data binning

**Figure 1:**
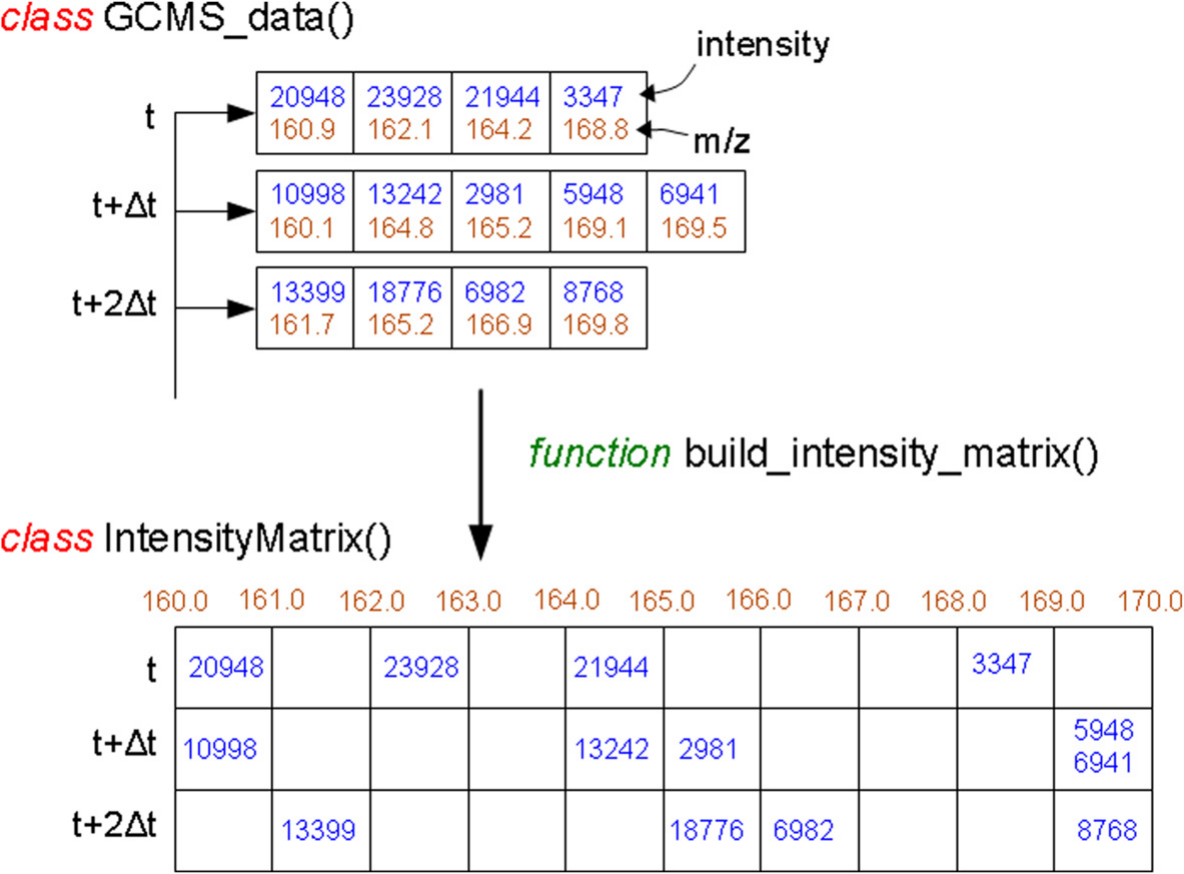
**The binning in PyMS.** The function "build_intensity_matrix()" performs binning on raw GC-MS data, and produces an object of the type "IntensityMatrix" from the object "GCMS_data" by binning. The object "GCMS_data" (holds the raw GC-MS data) consists of a list of mass spectral scans taken at equidistant time points along the retention time axis t, t + Δt, t + 2Δt , where each scan is a vector of (m/z, intensity) pairs. Binning creates a two-dimensional table whose cells are filled with sums of intensities that fall within each m/z bin. The "IntensityMatrix" object contains the binned data table, the vector of retention times that corresponds to the rows of the binned table (t = [t, t + Δt, t + 2Δt,…]), the vector of m/z which corresponds to the columns of the binned table (v = [ m_1_, m_2_, m_3_, … ]), and also defines a number of methods that operate specifically on the "IntensityMatrix" object. Empty cells of the binned table are filled with zeros (zeros not shown).

Raw GC-MS data stored by MS instruments consists of a series of mass spectral scans taken at equidistant time points along the retention time axis, where each scan is a vector of [m/z, intensity] pairs (Figure 1). The acquisition hardware associated computer typically performs centroiding on-the-fly, and as a result mass spectral scans have a non-uniform sampling in the m/z domain. Therefore the length of individual mass scan vectors may vary from scan to scan, and data in such arrangement is difficult to process. A widely accepted procedure is to bin the raw data to fixed m/z intervals [14], and this is particularly suitable for GC-MS data recorded at nominal mass resolution [6]. Quadrupole MS, most often used in GC-MS, generally acquire data at low resolution and the accurate mass of a metabolite can be slightly larger or slightly smaller compared to its nominal mass, depending on metabolites' exact atomic composition. Therefore, a concern in any m/z binning procedure is that chosen bin boundaries may be close to m/z of many observed metabolite fragments, as small inaccuracies in measurement may shift the fragment between different bins. A simple consideration of typical atomic composition shows that accurate mass of most metabolites is likely to be slightly larger than its nearest integer value. For this reason, commonly accepted boundaries for the unit-resolution binning are bins centered at around +0.2 to +0.3 of the unit mass, implying the bin boundaries near −0.3 (lower) and +0.7 (upper). This of course is a good choice only on average, and specific compound classes may have a different distribution of accurate masses (for example, a metabolite rich in oxygen and phosphorus may have accurate mass lower than an integer [6]). PyMS provides flexible binning functions that allow user to specify the desired binning parameters. By default, the data will be binned at nominal mass with bin boundaries set at −0.3 and +0.7, conforming to the behavior of most GC-MS data processing programs. However, user can specify different binning parameters by invoking optional arguments (example shown in Listing 4). The result of binning is always a "IntensityMatrix" object which has a number of special methods (e.g., a method that returns the size of the object, methods to return the minimum and the maximum mass resulted from binning, masses at bin centers, etc). In addition, the "IntensityMatrix" object also provides methods that can manipulate the data, for example crop mass spectral range between certain m/z values, or set intensities of certain masses to zero. The latter is useful for eliminating masses which are known to be uninformative (ie. artifact of the experimental procedure such are m/z = 73 and m/z = 147, commonly observed in TMS derivatized samples).

im = build_intensity_matrix_i(raw_data)

im = build_intensity_matrix(raw_data, 0.5, 0.25, 0.25)

**Listing 4.** Examples of building of the intensity matrix from raw data (data binning). In the above examples the variable "raw_data" is the "GCMS_data" object, and the variable "im" holds the binned data (assumed that that object "raw_data" was created as shown in the Listing 1). In the first command, the default binning is invoked (1 m/z unit intervals with bin boundaries set to −0.3 and +0.7). The second command shows an advanced binning (0.5 m/z interval units with bin boundaries set to −0.25 and +0.25). In PyMS binning of raw data results in an "IntensityMatrix" object, designed to capture and provide convenient access to binned data. The binned data takes a center stage in subsequent processing, and it is particularly important that the structure of this object is well suited for downstream data processing.

Conceptually, binned data can be viewed as a two dimensional matrix whose rows represent mass spectral scans, and columns represent ion chromatograms (ICs, reconstructed elution profiles of specific m/z species). Most of the subsequent processing workflow requires handling of individual rows and/or columns of this matrix. Elementary examples include time-domain noise smoothing of an IC (a column-wise operation), and mass spectral library matching (a row-wise operation). To allow efficient data handling, the "IntensityMatrix" object itself implements two kinds of objects: mass spectral scans ("MassSpectrum" type of object) and ICs ("IonChromatogram" type of object), and provides methods for manipulation of these objects (see Listing 5 for an example).

> > ms = im.get_ms_at_index(0)

> > ic = im.get_ic_at_index(0)

**Listing 5.** Examples of retrieving an individual ion chromatogram and mass spectrum from the intensity matrix object (binned data). In this example the variable "ms" is a "MassSpectrum" object, while the variable "ic" is the "IonChromatogram" object. These objects have their own attributes and methods that allow specific access to underlying data. It is assumed that that object "im" was created as shown in the Listing 4.

PyMS allows the binned data to be exported to three ASCII formats: as a space delimited two-dimensional matrix, as generic comma separated values (CSV) file, and as a Leco CSV file. These formats can facilitate exchange of binned data with external programs. For example, the space delimited CSV format allows import of a two-dimensional matrix into Matlab, while Leco CSV file is suitable for data import into other GC-MS processing software.

Most of the standard GC-MS data processing steps are performed by traversing the binned data either by mass spectra or by ion chromatograms. Combining the PyMS binned data structure with the general capabilities of the Python programming language allows one to build flexible yet powerful processing pipeline. For example, if the binned data is stored in the variable "im", each IC can be retrieved and processed as shown in Listing 6. An analogous loop would allow one to traverse through mass spectra instead of ion chromatograms (Listing 7).

n_scan, n_mz = im.get_size()

for i in range(n_mz):

 ic = im.get_ic_at_index(i)

 # do something with ion chromatogram

**Listing 6.** Looping over all ion chromatograms of binned data. It is assumed that that object "im" was created as shown in the Listing 4.

n_scan, n_mz = im.get_size()

for i in range(n_scan):

 scan = im.get_scan_at_index(i)

 # do something with mass spectrum

**Listing 7.** Looping over all mass spectra of binned data. It is assumed that that object "im" was created as shown in the Listing 4.

These examples show how to construct elementary data processing paradigms in PyMS. Additional flexibility is attained by combining PyMS data types with the general capabilities of the Python programming language. For example, if only certain ICs (e.g., certain m/z elution profiles) need to be processed, target ICs can be collected into a list (a Python native object), and the resulting list can be processed by looping over all items in the list (example not shown). This is an example of a data processing scenario that is simple to implement in PyMS, yet difficult to achieve with conventional GC-MS data processing software.

#### Noise filtering

Signals obtained from GC-MS instruments contain both high-frequency and low-frequency noise, and noise filtering is an essential step in routine processing of GC-MS data. The noise in GC-MS data can generally be divided into true noise (electronic noise) and chemical noise [30,43]. The representative true noise originates from limitations in instrument electronics (e.g., detector noise), and is typically manifested as high-frequency fluctuations with zero long-time average. On the other hand, chemical noise originates from chemical components introduced into the chromatography column/MS detector system, either during the sample preparation or because of instrument imperfections (such is column bleed) [43]. This type of noise often results in low-frequency signals, also known as baseline distortions. The detector noise is approximately constant within each mass trace (ion chromatogram), however, in practice the detector threshold is routinely applied resulting in blocks of zero intensities [24]. The nature of chemical noise is highly sample and method dependent; this kind of noise may manifest itself as a continuous, linear or irregular (e.g., "hump") contribution to the baseline. Therefore, in-depth analysis of noise in GC-MS experiments requires analysis of individual mass traces, taking into account blocks of zero data introduced by the detector threshold, and also analysis of noise as a function of time within each mass trace [24].

PyMS implements several methods for noise filtering. High-frequency noise filters ("noise smoothing") include moving-average and Savitzky-Golay filters [44]. Savitzky-Golay filters are based on least-squares polynomial smoothing, and this type of noise filtering is one the most commonly used in spectral data processing [45]. The moving-average filters are suitable for reducing random noise while retaining a steep response [46]. In PyMS, the width of the moving average window in both mean- and median-average filters is user controlled. With the Savitzky-Golay filter, both the widow width and the polynomial degree are user specified. As noise smoothing filters are applied to an "IonChromatogram" object, they can be applied to both individual mass traces and TICs. The application of a noise filter to an "IonChromatogram" object results in another object of the same kind, and therefore multi-pass filters can be easily applied.

Baseline distortion is often observed in GC-MS data (see for example [30]), and therefore baseline correction or low-frequency noise filtering is routinely required in GC-MS data processing. Approaches to baseline correction include a simple offset correction and methods that explicitly model the baseline (e.g. linear, cubic spline, or polynomial baseline correctors). Methods for baseline correction are also an active area of research, with several advanced methods reported recently, including the wavelet-based method developed for the electrophysiological data [47] and an automated parametric smoothing method developed for NMR [48,49]. PyMS implements an efficient baseline corrector based on mathematical morphology [50].

#### Peak object

The concept of signal peak is of fundamental importance in GC-MS data analysis [24,36,43], yet little discussion of data structures that can optimally support storage and handling of peak objects can be found in the literature. From the analytical perspective, the most important properties of a signal peak include peak elution time (retention time taken at the peak apex), peak mass spectrum (the mass spectrum taken at the peak apex), and the peak area (the sum of all m/z intensities between the left and right peak boundaries). In GC-MS, peak elution time and the peak mass spectrum are indicators of the component's chemical identity, while the peak area provides a quantitative measure of the component's abundance. In PyMS, a signal peak is stored in a special data structure ("Peak" object) that captures all important aspects of signal peaks, including peak retention time and peak mass spectrum, peak boundaries, and signal areas. It also assigns a unique ID to each peak at the time of peak object creation.

#### Peak detection and deconvolution

GC-MS is frequently applied for the analysis of complex samples resulting in heavy overlap of chromatographic peaks. This in turn poses significant challenges for the extraction of pure component mass spectra required for unambiguous component identification [43]. A number of methods for the extraction of pure component mass spectra from GC-MS data have been proposed in the past (see for example, [19,24,51-56]). These methods vary in their performance, accuracy, and suitability to specific features of GC-MS data, and to date no method has been accepted as standard [43]. PyMS implements peak deconvolution based on the ideas of Biller and Biemann [51], with several modifications. The PyMS peak deconvolution algorithm first identifies all local maxima that are above certain, user specified threshold. Then the neighboring apexing ions are combined, and initially considered to belong to the same signal peak. The allowed distance between the apexing ions to combine into the same pure component, the intensity threshold and the width of the window over which local maxima are detected are all user adjustable parameters. PyMS allows two types of signal peak quantitation: all ions found to belong to a single component can be used for quantitation; alternatively, a specific ion can be automatically selected for consistent quantitation when experimental replicates are available (see below the section "Single ion quantitation"). To calculate the area of a single m/z trace (i.e. a single component of a multi-component peak), the intensities are added from the apex of the mass peak outwards. Edge values are progressively added until the added intensity contributes less than a certain threshold relative to the accumulated intensity (default = 0.5%), or the intensities start to increase. This prevents the accumulation of intensities from the neighboring signals, as the increase in intensities is an indication that the overlapping signal is present.

#### Peak alignment by dynamic programming

When a number of related GC-MS experiments are analyzed it is essential to account for retention time drifts between individual runs [27,30,31,36,57-59]. This is typically achieved either by aligning chromatographic profiles prior to peak detection (see for example, [58,60,61]) or by matching signal peaks between the experimental runs post-peak detection [27,36,57]. PyMS implements a post-peak detection alignment approach based on dynamic programming (DP), described in detail elsewhere [36]. Here we briefly review only the most fundamental aspects of the DP peak alignment. The best alignment is achieved by minimizing the alignment score by DP, where the individual peak-to-peak scoring function is chosen to take into account similarities in both peak retention times and mass spectra [36]. The final alignment is built progressively, by adding one experiment at a time, based on the guide-tree derived from all possible pairwise alignments [36]. The final peak alignment table relates signal peaks obtained in individual experimental runs across the entire set of experiments and this may involve a set of replicate experiments on a single biological state, or several biological states each characterized by a number of replicate experiments. In the final alignment matrix the positions of individual peaks are replaced with respective peak areas that quantify the concentrations of individual metabolites. This method can be used to align peaks across large numbers (e.g., hundreds) of GC-MS runs.

#### Single ion quantitation

**Figure 2:**
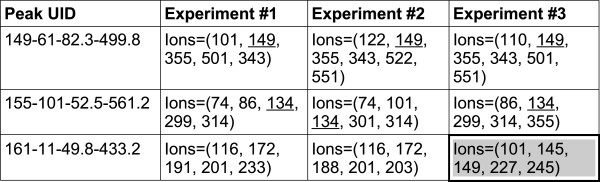
**The single ion quantitation algorithm as implemented in PyMS.** Shown is a hypothetical alignment table with three peak positions (peak UIDs "149-61-82.3-499.8", "155-101-52.5-561.2", and "161-11-49.8-433.2"), with nine individual peaks detected in three different experiments (shown as columns). For each individual peak, PyMS keeps track of the full mass spectrum and all m/z ions from the peak begin and end scans. In the single ion quantitation algorithm, the N most intensive ions are extracted for each peak (by default, N = 5). For each peak position, a single common ion present in all peaks is found. If multiple such ions may exits, the first found is used. This procedure aims to find at least one that will be used for consistent quantitation at this peak position across all experiments. In the example shown, for the first and second peak the selected common ions are m/z = 149 and m/z = 134, respectively (underlined). For the third peak position (UID = "161-11-49.8-433.2"), none of the ions present in both experiments #1 and are found in the top five ions of the experiment #3, suggesting the misalignment for this peak position (peak cell highlighted).

A common scenario in metabolomic GC-MS studies is non-targeted analysis of multiple (possibly many) experimental runs. When many signals are considered across a number of experimental runs, and a prior knowledge of component mass spectra is not available, the key challenge is to ensure that each peak is quantified with exactly the same set of ions. A common approach to this is to base the quantitation on a single ion, and then use the selected ion consistently across different samples. This works well when characteristic ions for compounds of interest are known beforehand (i.e. in targeted metabolomic studies). In non-targeted studies characteristic ions may not be known for all components beforehand. To address this we have developed an approach that automatically selects an ion for quantitation across all samples for each unknown peak (Figure 2). The starting point for non-targeted single ion quantitation is the peak alignment table. It should be noted that the alignment table produced by DP incorporates the full peak mass spectra taken at the peak apexes. Then, as a part of single ion quantitation algorithm, the most *N* abundant ions are selected for each peak position, and the peaks in the alignment table are examined to find a single ion common to all peaks aligned at that position. For each peak, the area of this single ion is integrated across the retention time limits determined by the peak area calculation algorithm described above and used for quantitation across multiple samples. This approach achieves a consistent quantitation in multiple-experiment scenarios where the compound characteristic ions are not known beforehand (Figure 2). Since this approach relies on the most abundant m/z ions for each peak, it excludes any noise arising from other m/z channels, as well as interference from neighboring peaks. In addition, this approach implicitly checks the validity of the peak alignment table: if the quantitation ion is not present in a specific occurrence of the peak, the peak is probably misaligned.

### Design and implementation

#### Organization of the PyMS project

The PyMS project consists of three related parts hosted as independent projects on the publicly accessible Google Code repository, and test data repository hosted on our own servers (Table 1). The Google Code repositories includes a source code repository ("pyms" project), an extensive set of ready to run tests and examples ("pyms-test" project), and User Guide ("pyms-docs" project).

**Table 1:** The summary of PyMS projects, and the URLs for public access

**Project name**	**Project URL**	**Explanation**
pyms	http://code.google.com/p/pyms/	PyMS code
pyms-test	http://code.google.com/p/pyms-test/	Tests & ready-to-run examples
pyms-docs	http://code.google.com/p/pyms-docs/	PyMS documentation

The project "pyms-test" contains 42 ready-to-run scripts that illustrate all aspects of current PyMS functionality. Because of space restrictions on the Google Code public Web server, the example data sets used by the test scripts in "pyms-test" are provided separately, and can be obtained from http://bioinformatics.bio21.unimelb.edu.au/pyms/data/.

#### Modularity

PyMS is implemented as a hierarchy of Python sub-packages, allowing any functionality to be selectively invoked as required. The overall architecture of PyMS is explained in the User Guide. The individual PyMS functions focus on specific, well-defined processing tasks, while each function is designed to be as flexible as possible by using optional and named arguments to handle optional parameters. To illustrate this, consider the Savitzky-Golay filter module. This module is represented by the file "SavitzkyGolay.py" which defines the function "savitzky_golay()" (see Listing 8). To achieve Savitzky-Golay filtering one needs to load the "savitzky_golay()" function from the PyMS package hierarchy at runtime, and then apply the function to a suitable object. The user is able to change function parameters by specifying the optional, named arguments on the command line. This example illustrates the general principles used throughout the PyMS implementation.

def savitzky_golay(ic, window = DEFAULT_WINDOW, \

 degree = DEFAULT_POLYNOMIAL_DEGREE)

**Listing 8.** The definition of the function "savitzky_golay" within the module "SavitzkyGolay.py". The argument "ic" is the ion chromatogram passed to the function, while the named parameters "window" and "degree" are optional. If the optional named parameters are not specified when in the function call they take default values specified above.

#### Parallelization

A typical GC-MS data may consist of 3,000-10,000 mass spectral scans with ~500 m/z values collected in each scan. Standard GC-MS data processing involves processing of series of ICs or mass spectra, as iterative column-wise (ICs) or row-wise (mass spectra) processing of the binned intensity matrix. PyMS implements parallel processing based on MPI (Message Passing Interface), a language-independent communications protocol for parallel processing. PyMS uses MPI for Python (mpi4py), a Python package that provides bindings of the MPI standard. At present PyMS is able to harvest multiple CPUs only for by-row and by-column data processing tasks (for example, IC noise filtering and baseline correction steps).

## Methods

### Sample preparation

#### Metabolite mix analysis by GC-MS

A mixture of 45 metabolites representing a variety of chemical (sugars (0.1 mM), organic acids, amino acids, sugar phosphates (1 mM)) was prepared for GC-MS analysis. Specifically, 10 μl of the mixture was transferred to a GC-MS vial insert and evaporated to dryness in vacuo. Samples were methoximated in 20 mg/ml methoxyamine in pyridine (20 μl, 16 hr, 25°C) with continuous shaking, and then derivatized with BSTFA + 1% TMCS (Pierce; 20 μl, 1 hr, 25°C) using a Gerstel MPS2 autosampler robot. Samples (1 μL) were injected onto an Agilent 7890A GC interfaced with a 5975 C mass selective detector. GC was performed on a 30 m VF5-MS column with 0.25 mm inner diameter and 0.25 mm film thickness (Varian Inc.). Injection temperature was 250°C, the interface set at 280°C, and the ion source adjusted to 250°C. The carrier gas was helium (flow rate 1 ml/min). The temperature program was 1 min isothermal heating at 70°C, followed by a 1°C/min oven temperature ramp to 76°C, then 5°C/min to 325°C and held for 10 min. Mass spectra were recorded at 2.66 scans/s (m/z 50-600).

Foetal calf serum analysis by GC-MS. Foetal calf serum samples were spiked with 2-fold increasing amounts of a mix of metabolite standards (Final concentration: sugars (0.78 – 12.5 μM), organic acids (3.9 - 62.5 μM), amino acids (7.8 – 125 μM)). Samples (120 μL) were extracted by the addition of 200 μL ice-cold chloroform:methanol (3:2 v/v), followed by vigorous mixing and incubation on ice for 10 min. Samples were centrifuged at 4°C for 10 min at 16,000 rpm to enable biphasic separation. The upper aqueous phase (15 μL) was transferred to GC-MS vial inserts and dried *in vacuo*. Samples were prepared for GC-MS analysis as above and injected (1 μL). GC-MS settings were as above, but temperature program was 2 minutes isothermal heating at 35°C, followed by a 25°C/min oven temperature ramp to 325°C and held for 5 minutes. Mass spectra were recorded at 9.19 scans/s (m/z 50–600).

### Data analysis

A comparative data analysis was performed with four software packages: AnalyzerPro (SpectralWorks, Runcorn, United Kingdom), AMDIS [24], XCMS [15], and PyMS (this work). Firstly, the two data sets described above (metabolite mix and foetal calf serum with spiked metabolites) were analyzed with a combination of these packages and with the assistance of experienced analysts to determine the number of components in each data set. Subsequently, each software package was applied to each data set. For each software package optimization of processing parameters was performed independently by comparing the results with the manually determined component content. Although certain processing parameters are similar between these software packages, no effort was made to make them consistent between the packages. This is because other, non-similar parameters also influence the quality of results. Therefore we have focused on optimizing the parameters for each package in absolute terms, by comparing the results to the manually determined components. Three out of four compared software packages (AnalyzerPro (SpectralWorks, Runcorn, United Kingdom), AMDIS [24], and PyMS, but not XCMS [15]) give the option to ignore the ion chromatograms (traces of intensities over time) of certain ions, allowing the user to introduce prior knowledge about the experimental setup to improve data analysis. As the EI mass spectrum of TMS derivatized metabolites contain the ions m/z = 73 and 147, these ions were ignored in the analysis by AnalyzerPro (SpectralWorks, Runcorn, United Kingdom), AMDIS [24], and PyMS.

#### AMDIS

AMDIS is most frequently used in connection with the NIST database of metabolites for detection of compounds in GC-MS data. In this study, only the peak detection ability of AMDIS was tested, with no evaluation of its library matching ability. AMDIS parameter 'Shape requirements', used for shape matching in the peak detection algorithm was set to 'Medium'. Sensitivity was also set to 'Medium', and the 'Type of analysis' parameter was set to 'Simple'. AMDIS has a spectral resolution parameter 'Adjacent Peak Subtraction' and this was set to 1. The component width was set to 32, and this was found to give the best results for analysis of both the metabolite mix and spiked foetal calf serum.

#### XCMS

We used the XCMS 'Matched Peak' method [15]. The signal to noise ratio must also be set, and a value of 5 was found to give the best results. The 'FWHM' (full width at half maximum) parameter was set to 0.01 minutes.

#### AnalyzerPro

AnalyzerPro is a commercial, specialized GC-MS data tool (SpectralWorks, Runcorn, United Kingdom). Many of AnalyzerPro data processing parameters can be set from knowledge of the raw data acquisition parameters. Manual optimization suggested slightly different parameters for the two data sets (metabolite mix and foetal calf serum) to give optimal results. In the processing of metabolite mix, the following parameters were used: area threshold = 1000, ions = 3, scans = 2, height threshold = 1, signal to noise = 5, smoothing = Gaussian 5 points, width threshold = 0.06 minutes. In the processing of foetal calf serum data, the following parameters were used: area threshold = 500, ions = 2, scans = 3, height threshold = 1, signal to noise = 5, smoothing = Gaussian 5 points, width threshold = 0.01 minutes.

#### PyMS

Although PyMS has many parameters which can be set by the user, only five parameters were used for optimization of peak finding in the processing of metabolite mix and foetal calf serum data. Other parameters (e.g., Savitzky-Golay noise filtering parameters and window filtering parameters) were left at default values. The five parameters optimized for peak detection in PyMS were: window width (the window over which local maxima are detected in initial peak finding), threshold (a minimum intensity that a certain number of ions, set by the parameter "ions"*,* must have in order to remain a peak during filtering of the peak list), ions (the minimum number of ions with intensity above "threshold" which a peak must possess in order to remain in the peak list after filtering), scans (the spectral resolution parameter; ions with local maxima separated by "scans" number of time points/scans will be included in the same peak object), and r (this parameter is used to remove ions in the peak object which are due to low level noise. Expressed as a percentage, any ions with intensity below *r%* of the base ion of the peak will be removed from the compounded GC-MS peak). For the processing of metabolite mix data, the following parameters were used: window = 5, ions = 3, scans = 2, threshold = 2000, r = 2%. For the processing of foetal calf serum data, the following parameters were used: window = 13, ions = 3, scans = 2, threshold = 6000, r = 2%.

## Results

We performed comparative analysis of PyMS with several well established software packages, on in-house generated data under different conditions and experiments specifically designed for this purpose. The software packages included in the comparative analysis were AMDIS [24], XCMS [15], and AnalyzerPro (SpectralWorks, Runcorn, United Kingdom). Two different experiments were designed to generate the optimally informative data: a mix of 45 metabolites representing a variety of chemical classes (sugars, organic acids, amino acids, sugar phosphates), and foetal calf serum spiked with metabolite standards, representing a sample with complex biological matrix (see Methods). Each data set was scrutinized manually by analytical experts, and the signal peaks were manually picked and deconvoluted. The results of manual analyses served as the best approximation of the desired answer against which we benchmarked the automated peak detection. All four software packages (AMDIS, XCMS, AnalyzerPro, and PyMS) were applied starting from the raw data, and for each software package an effort was made to optimize the processing parameters so to obtain the closest result relative to the manually obtained results (see Methods).

**Figure 3:**
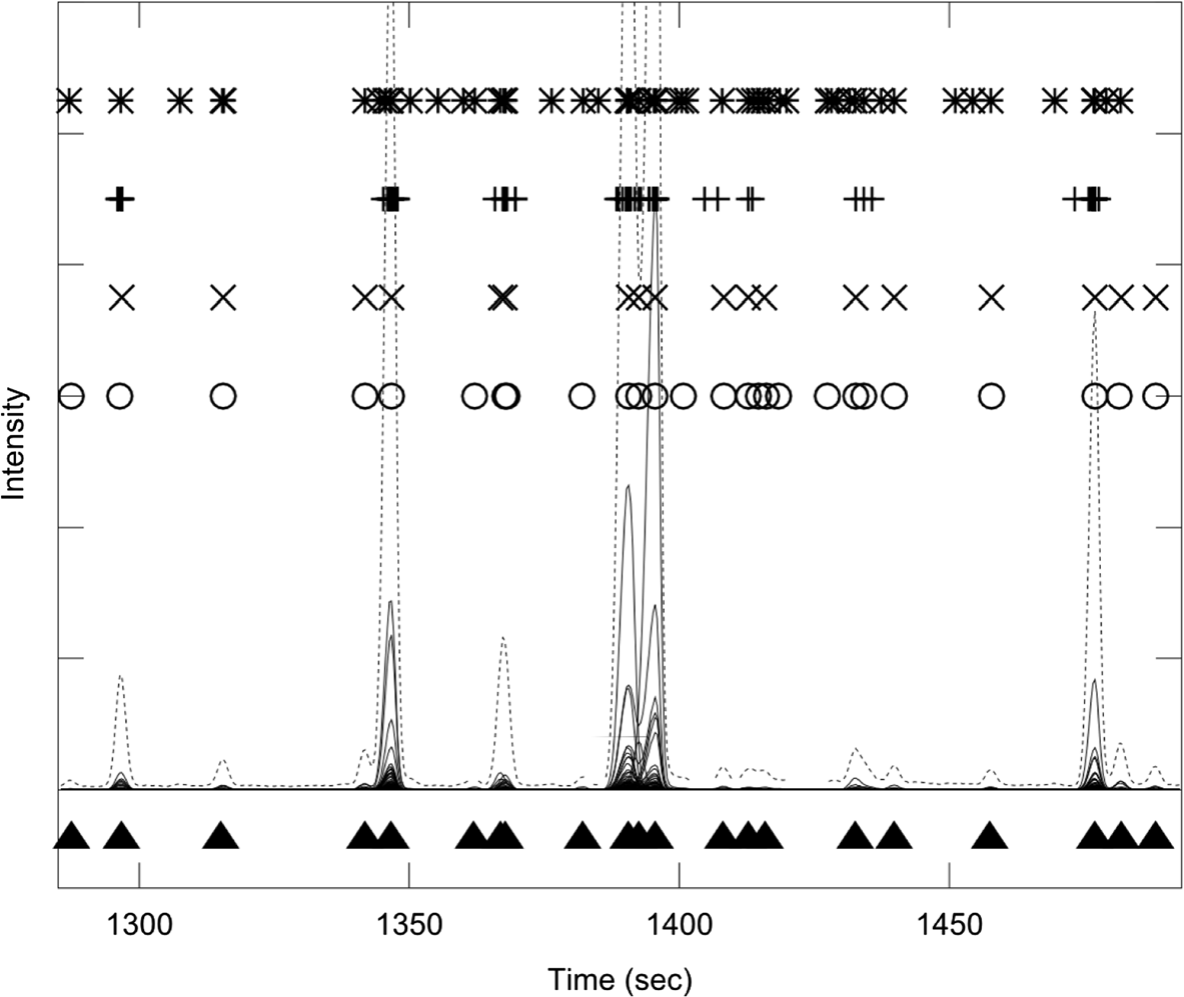
**The experiment with low complexity sample matrix and well resolved peaks.** A segment of a GC-MS experiment recorded on a control mix with 45 reference compounds. The total ion chromatogram (TIC) is shown in dotted line, and individual ion chromatograms (ICs) in the m/z range 50-550 are overlaid in full line. The true multi-component GC-MS peaks, identified manually by an experienced operator, are shown as filled triangles at the bottom of the graph. The peaks detected by four software packages are shown in the upper part of the Figure ('*'― AMDIS, ' + ' ― XCMS, 'x' ― PyMS, 'o' ― AnalyzerPro). The segment chosen for this analysis is sparsely populated with peaks, which are all well resolved. All four software packages performed comparably to the human operator. Clustering of individual single ion peaks around true multi-component GC-MS peaks is evident from the peak detected by XCMS. For the list of detected signals see Additional file 1.

Figure 3 shows a segment of the total ion chromatogram (TIC) from the metabolite mix experiment which contains a number of well resolved chromatographic peaks. In addition to TIC (dotted line), all individual ICs are shown (solid line), to make clearer the location of individual signal peaks. Filled triangles below the TIC represent manually annotated signal peaks by an expert human operator. Although there are some differences, the performance of different software packages was comparable on this data segment, and matched closely the manual annotation. Some tendency of AMDIS to report false positives is evident, as reported before [62].

**Figure 4:**
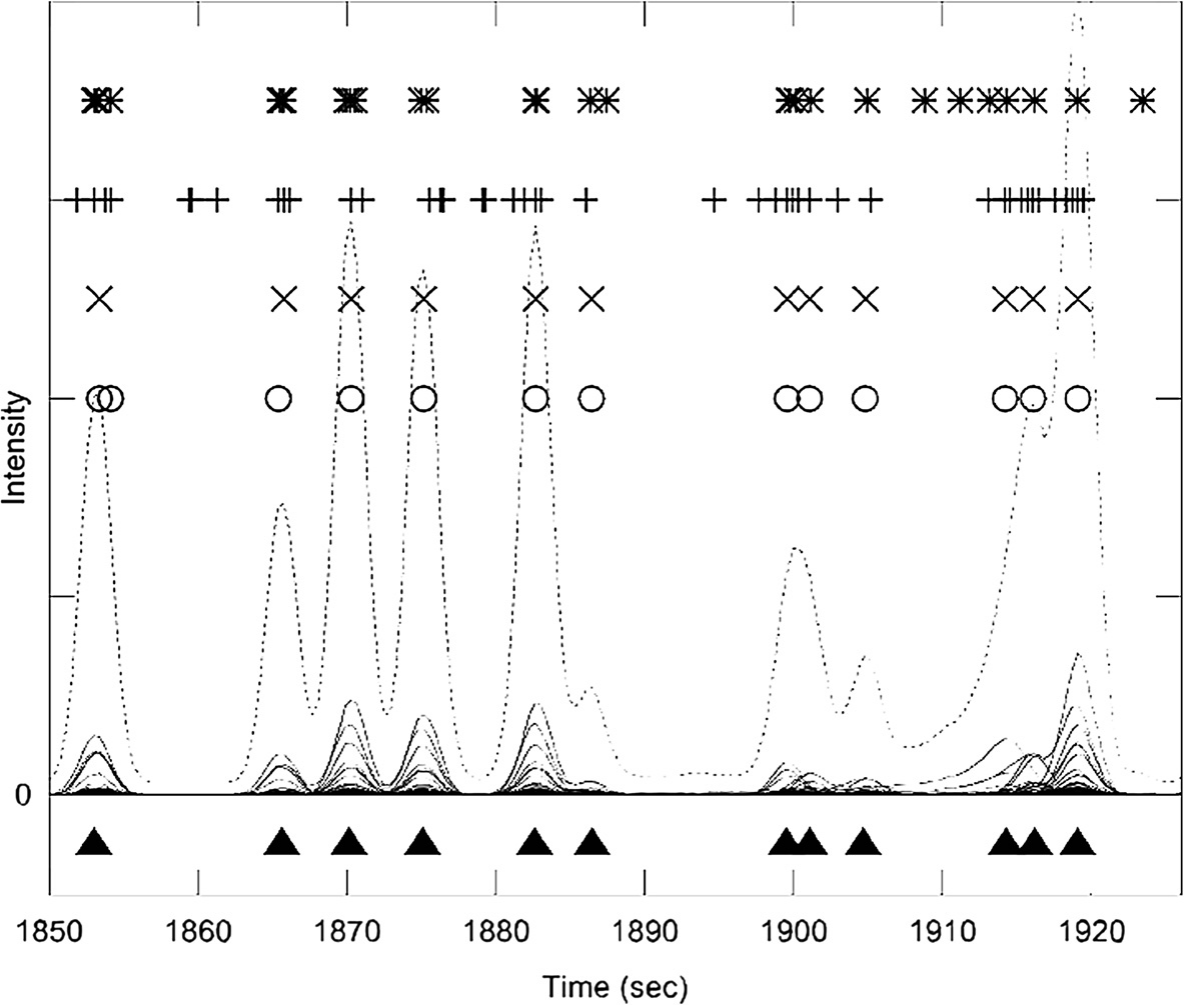
**The experiment with low complexity sample matrix and moderate peak overlap.** A segment of a GC-MS experiment recorded on a control mix with 45 reference compounds. The total ion chromatogram (TIC) is shown in dotted line, and individual ion chromatograms (ICs) in the m/z range 50-550 are overlaid in full line. The true multi-component GC-MS peaks, identified manually by an experienced operator, are shown as filled triangles at the bottom of the graph. The peaks detected by four software packages are shown in the upper part of the Figure ('*'― AMDIS, '+' ― XCMS, 'x' ― PyMS, 'o' ― AnalyzerPro). This elution segment includes few closely eluting peaks in the retention time range 1895-1920 s. For example, the range 1910-1920 s contains three distinct co-eluting compounds to give appearance of two TIC peaks. Both PyMS and AnalyzerPro deconvoluted these three peaks successfully, and also detected the other peaks in the chromatogram comparably to the human operator. AMDIS significantly overestimated the number of peaks on this segment. By default, XCMS looks for single ion peaks and not GC-MS type multi-ion peaks, and therefore in this segment detected many such peaks. For the list of detected signals see Additional file 2.

Figure 4 shows a different segment of the same data set (metabolite mix). In addition to some well resolved peaks, this segment contains several moderately overlapped peak clusters in the retention time range 1900-1930 s. The highly accurate peak detection by PyMS and AnalyzerPro is evident. Both AMDIS [24] and XCMS [15] detect signals accurately, but also report a number of additional signals. A closer scrutiny of the results shows that the underlying reason for this is different for the two software packages. XCMS has been primarily developed for the processing of LC-MS data [15], and does not make an attempt to assemble single ion signals into fragmentation mass spectra characteristic of GC-MS data generated with electron impact (EI) ionization. On the other hand, AMDIS has been developed specifically for EI-GC-MS data and additional signals reported by AMDIS are therefore false positives.

**Figure 5:**
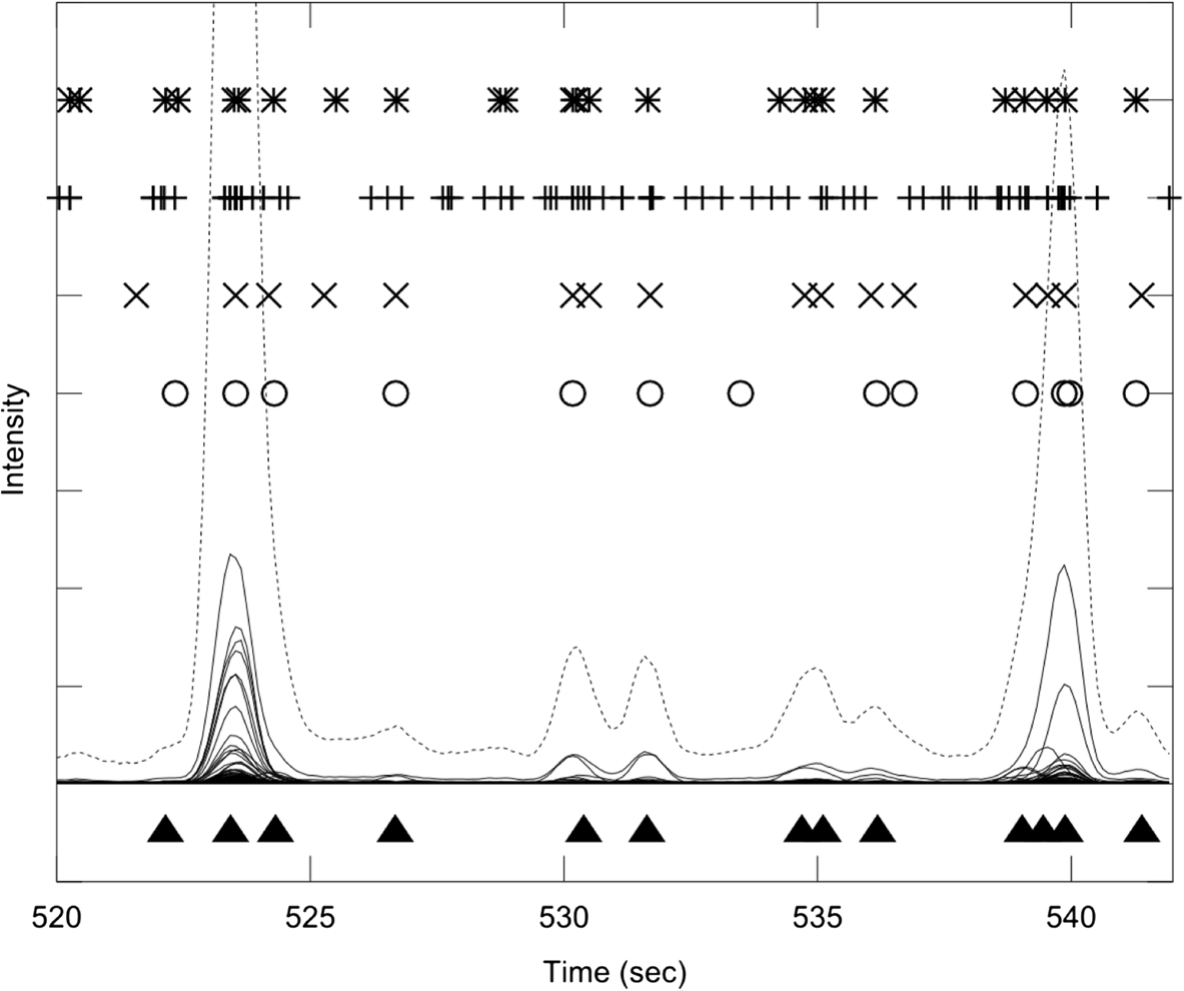
**The experiment with complex biological matrix and moderate peak overlap.** A GC-MS experiment was recorded on foetal calf serum spiked with a mix of 17 known compounds. The total ion chromatogram (TIC) is shown in dotted line, and individual ion chromatograms (ICs) in the m/z range 50-550 are overlaid in full line. The true multi-component GC-MS peaks, identified manually by an experienced operator, are shown as filled triangles at the bottom of the graph. The peaks detected by four software packages are shown in the upper part of the Figure ('*'― AMDIS, ' + ' ― XCMS, 'x' ― PyMS, 'o' ― AnalyzerPro). The shown region includes two of the spiked compounds, aspartic acid (the large peak near 524 s) and malic acid (the large peak near 540 s). Two smaller peaks are closely eluting with malic acid providing a good test for deconvolution. Both PyMS and AnalyzerPro performed similarly to an experienced operator in the identification of peaks. For the list of detected signals see Additional file 3.

Figure 5 shows a short segment of the data obtained on metabolite standards spiked into foetal calf serum to generate a complex biological background matrix. In this example AMDIS performed well, and has accurately detected most signal peaks (note the signal clusters near 523 s and 540 s). In a situation exacerbated by the complex biological matrix, XCMS reported a large number of single ion signals. Overall, the peaks detected by PyMS and AnalyzerPro were the closest to the signals as annotated by the human operator. Both AnalyzerPro and PyMS failed to detect accurately several signals peaks: AnalyzerPro reported two false positives and failed to detect two peaks clearly visible near 535 s, while PyMS reported three false positives, including a weak signal near 531 s visible from the ion traces.

**Figure 6:**
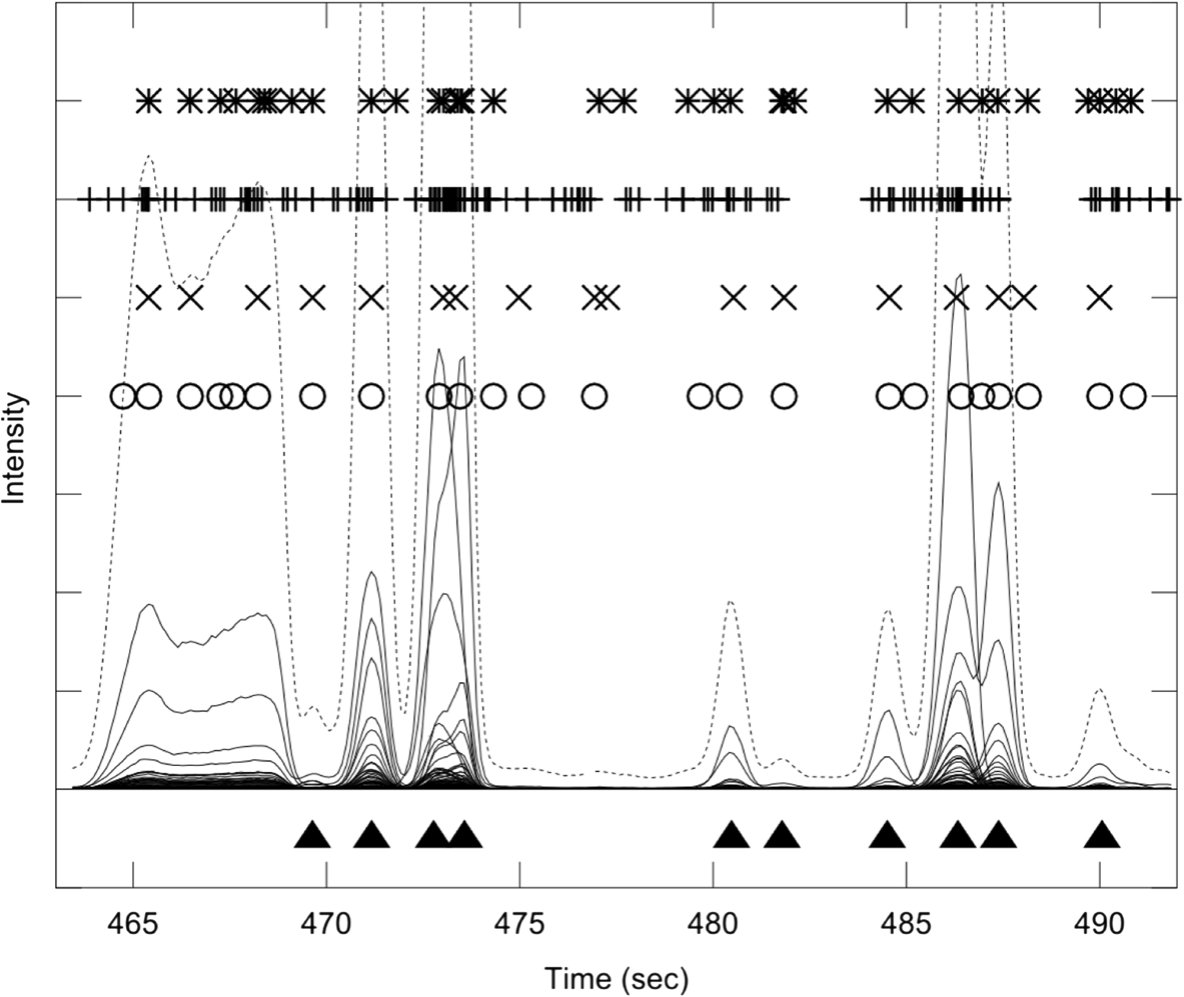
**The experiment with complex biological matrix and heavy peak overlap.** A GC-MS experiment recorded on foetal calf serum spiked with a mix of 17 known compounds. The total ion chromatogram (TIC) is shown in dotted line, and individual ion chromatograms (ICs) in the m/z range 50–550 are overlaid in full line. The true multi-component GC-MS peaks, identified manually by an experienced operator, are shown as filled triangles at the bottom of the graph. The broad peak observed in the retention time range 464-469 s is an overloaded urea peak. The four packages have each picked different numbers of peaks, with AMDIS and XCMS picking many more peaks than either PyMS or AnalyzerPro. Both PyMS and AnalyzerPro performed similarly to an experienced operator, with some false positive peaks reported. In the area near 476 s both software reported several weak signals, not annotated by the human operator. For the list of detected signals see Additional file 4.

Figure 6 shows an extremely complex case obtained on foetal calf serum sample spiked with metabolite standards, involving both a heavy peak overlap and a complex biological matrix. Neglecting the broad signal 464–468 s (originating from column overloaded urea), AnalyzerPro reported seven false peaks in the region >468 s, and was outperformed by PyMS which reported only three false peaks. We note that in this example both PyMS and AnalyzerPro reported several peaks in the area near 476 s, not annotated by the human operator.

Quantitation in metabolomics can be either relative or absolute: relative quantitation is typically used in non-targeted metabolic profiling, while absolute quantitation is most often used in targeted studies. In relative quantitation the signal intensities are normalized by another signal observed within the same experiment (typically a suitably chosen metabolite or externally added compound), or with a suitable function of several signals (e.g., median observed over a certain chromatographic segment). In absolute quantitation the samples are spiked with reference compounds of known concentration prior to the GC-MS run [63].

**Figure 7:**
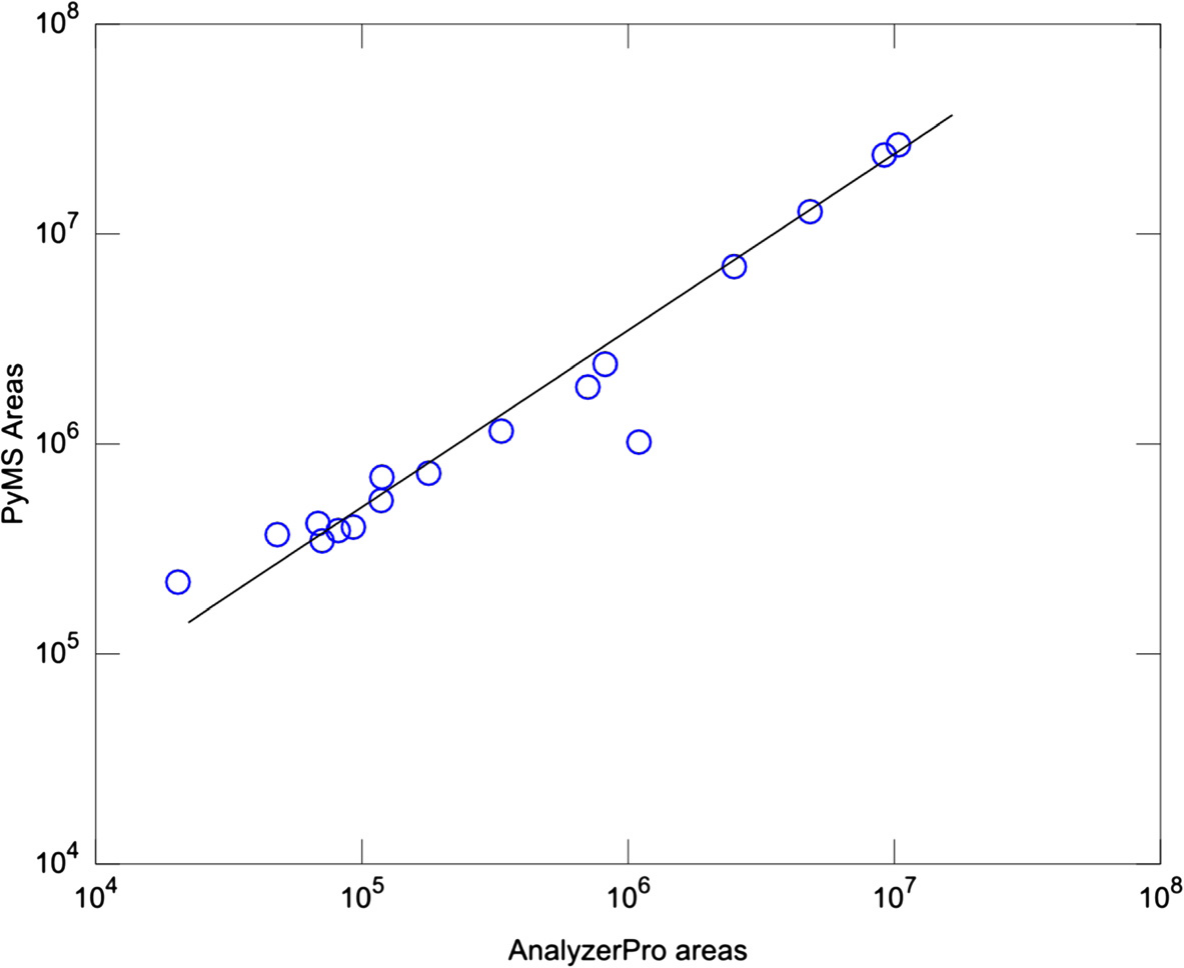
**Relative quantitation by AnalyzerPro and PyMS.** Shown is a comparison of raw areas calculated by AnalyzerPro (x-axis) and PyMS (y-axis) for the data and segment depicted in Figure 3, including signal peaks correctly identified by both programs. The segment chosen for this analysis involves largely well-resolved peaks, and therefore provides a good test of relative quantitation without interference with potential errors originating from deconvolution. For each program its own internal algorithm was used to calculate peak boundaries and total peal areas. A good linear relationship in raw areas reported by two software packages was observed. The correlation coefficient for the data shown is 0.998.

The assessment of software quantitative capabilities requires analysis over a range of concentrations in a specifically designed set of experiments. We have chosen to focus on PyMS and AnalyzerPro for the assessment of quantitative capabilities. To assess relative quantitation, experiments with 45 compounds metabolite mix were used because of well resolved peaks and low-complexity background matrix. Both the linearity and correlation in raw areas, as reported by PyMS and AnalyzerPro, was assessed. The peaks detected by both programs on the segment shown in Figure 3 were selected (a total of 17 peaks), and peaks were quantitated by both AnalyzerPro and PyMS. This particular segment is populated with well-resolved peaks thereby minimizing any contributions from errors that may be introduced by peak deconvolution algorithms. A good linear relationship between the results was obtained by two software packages (Figure 7), with the correlation coefficient of 0.998.

**Figure 8:**
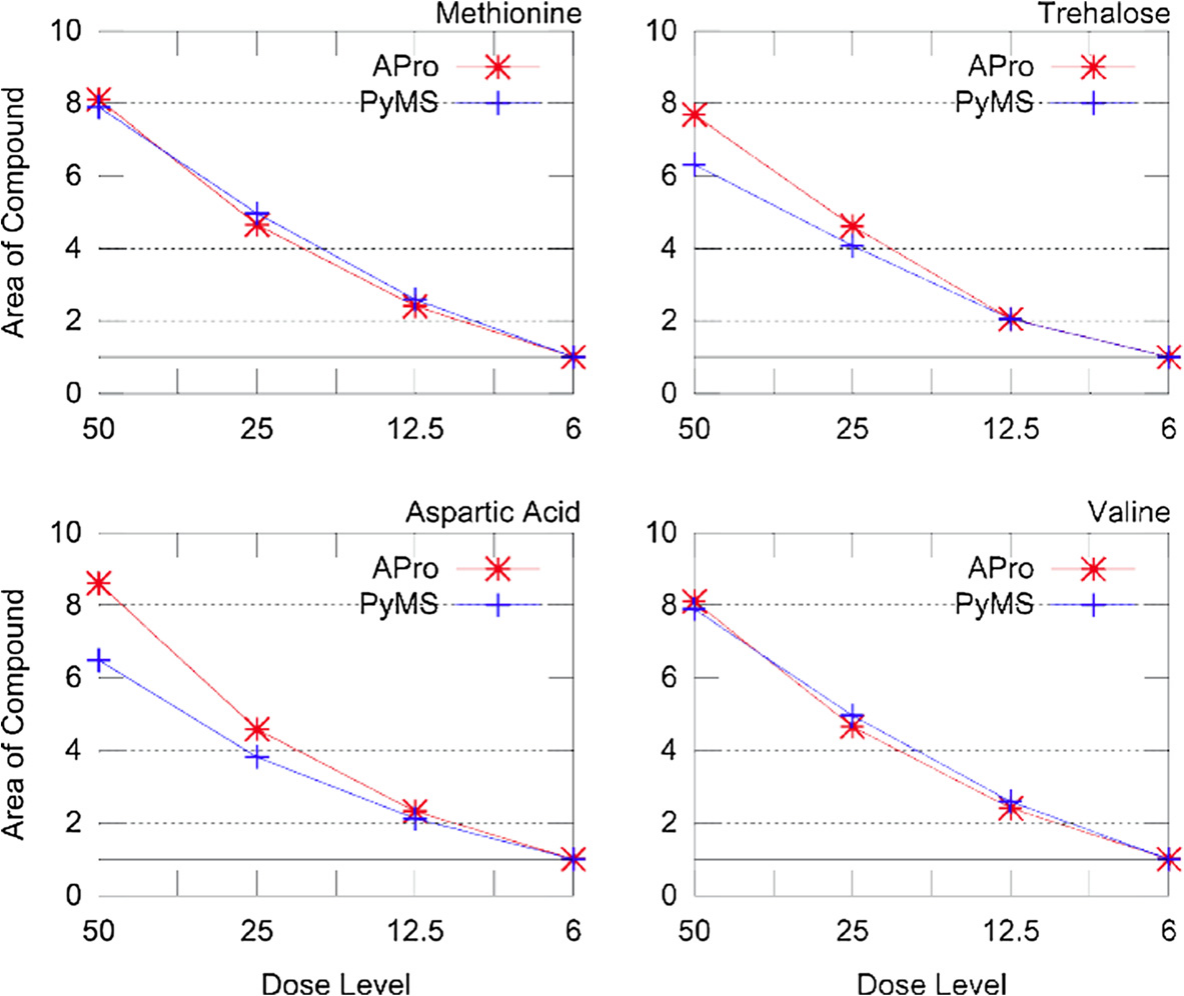
**Absolute quantitation with PyMS and AnalyzerPro.** The samples of foetal calf serum were spiked with a decreasing concentration of a mix of 17 reference compounds. Shown is the quantitation of peak areas by PyMS and AnalyzerPro for four different reference compounds (methionine, trehalose, aspartic acid, and valine), across four different GC-MS experiments, spiked with 6, 12.5, 25, and 50 μl of reference compounds (the dose level of spiked reference compounds is shown as a sample label on the x-axis). To account for metabolites naturally occurring in foetal calf serum, the areas were normalized to the lowest concentration of each compound. A good agreement in expected and observed absolute quantitation was observed for both AnalyzerPro and PyMS.

To assess absolute quantitation, experiments that involved spiking quantitative amounts of reference compounds in foetal calf serum were analyzed. Figure 8 shows quantitation results for four reference metabolites, namely methionine, trehalose, aspartate, and valine. In these experiments metabolites were spiked in two-fold increasing concentrations, thereby allowing one to assess absolute quantitation. When ordered by decreasing concentrations of the reference compounds, successive experiments are expected to have half the concentration of the previous experiment. A good agreement between results obtained by AnalyzerPro and PyMS was observed. Furthermore, the expected fold-increase in spiked reference compounds was closely reproduced (Figure 8).

## Discussion

It is estimated that about half of published GC-MS metabolic profiling studies is dedicated to methods development [5]. A substantial portion of this is relevant to various aspects of data processing, analysis, and information management (e.g., development of mass spectral databases). A recent surge in academic work focusing on GC/LC-MS data analysis has significantly increased available software tools, providing a variety of choices for raw GC/LC-MS data processing [15-19,30,31,35,64]. In spite of this, the field of GC-MS data processing remains highly dynamic, and new tools are likely to profoundly influence the field and established processing protocols.

Here we report the development of PyMS, a library of functions for processing of GC-MS data developed in Python. Python is an object-oriented, general purpose scripting language widely used in scientific applications, that is gaining attention of the MS data processing community [34,35]. PyMS currently provides a complete set of GC-MS processing functions, including reading of standard data formats (ANDI-MS/NetCDF and JCAMP-DX), noise smoothing, baseline correction, peak detection, peak deconvolution, peak integration, and peak alignment by dynamic programming described previously [36]. A novel common ion single quantitation algorithm allows automated, accurate quantitation of GC-MS electron impact (EI) fragmentation spectra when a large number of experiments are analyzed (see Methods). PyMS implements parallel processing based on Message Passing Interface (MPI), and is able to harvest multiple CPUs for by-row and by-column data processing tasks (for example, noise filtering and baseline correction steps), allowing processing to scale on multiple CPUs in distributed computing environments.

Here we report a comparative evaluation of PyMS peak detection and quantitation, together with three well established software packages (AMDIS [24], XCMS [15], and AnalyzerPro (SpectralWorks, Runcorn, United Kingdom)). For this purpose a specific set of experiments was designed, and we used experimental data acquired in-house. For each software package the parameters were optimized by comparing the results to manual peak detection performed by an experienced analyst familiar with the samples and experimental design. In peak detection and deconvolution PyMS and AnalyzerPro achieved overall the most consistent results with manual analyses. We note that in a recently reported comparative study of GC-TOF-MS data, AnalyzerPro outperformed AMDIS and a commercial software package ChromaTOF (Leco Corporation, St. Joseph, MI) [62].

The observed performance of AMDIS and XCMS can be consolidated with the reported literature. XCMS was primarily developed for the processing of LC-MS data, and is highly tuned to the information content of LC-MS data [15]. XCMS looks for signal peaks on individual m/z traces by finding signal maxima within 0.1 m/z slices, matched filtration, and signal-to-noise ratio cutoff [15]. Consistently, in our studies XCMS accurately detected signals in single GC-MS ion traces, but without an attempt to assemble individual ion signals into fragmentation mass spectra characteristic of GC-MS with EI ionization. This is the main reason for the large number of signals detected by XCMS (Figures 4, 5, 6, 7). We note that this may have implications for several recent studies that used XCMS for GC-MS data processing [65-69].

In contrast to XCMS, AMDIS was specifically developed for the processing of GC-MS data [24]. The tendency of AMDIS to overestimate the number of signals was reported previously [25,62]. In a comparative study of GC-TOF-MS data, AMDIS reported up to 70–80% artefactual components [62]. We note that recently developed complementary software packages assist in data correction and filtering required when processing data with AMDIS [25,26]. These complementary software packages are relatively new, and therefore the native capabilities of AMDIS as used in our study account for the vast majority of AMDIS applications.

PyMS specifically aims to decouple processing methods from visualization, and to expose processing methods through the command line interface. Therefore the main strength of PyMS is non-interactive GC-MS data processing, where commands are packaged into scripts and executed in the batch mode. PyMS can also be used in interactive data processing and exploratory data analysis, and provides limited graphical capabilities (for example, allowing user to plot individual ion chromatograms and mass spectra). A number of MS software packages with advanced graphical interfaces exist, providing a high complementarity. Often, the aim of intuitive use dictates a high level of integration between the processing methods and GUI components [16-19,24,30]. While GUI-based software remains highly important in MS data processing, command line tools provide advantages in certain scenarios. For example, a previously developed processing pipeline is much easier to apply in a repetitive fashion with suitable command line tools. Furthermore, a decoupling of processing methods from GUI allows implementation of additional algorithms without the need to address (often complicated) interaction of processing methods with GUI components. This in turn facilitates rapid prototyping and implementation of new processing algorithms.

PyMS is essentially a library of processing functions implemented in Python (http://www.python.org/), and in this regards is similar to XCMS, a library of processing functions implemented in R (http://www.r-project.org/). While R is an integrated, interactive environment for statistical analysis, Python is a general-purpose programming language. The latter is an obvious advantage when general programming problems are involved, as is the case in many aspects of MS data processing and analysis (e.g. file parsing, signal processing, the need to use web services). However, in later stages of most processing/analysis workflows statistical analysis becomes important. Because PyMS operates fully within the Python environment, statistical capabilities of R can be integrated with the PyMS capabilities through a suitable Python interface (for example RPy, http://rpy.sourceforge.net/).

Two aspects not addressed in the current work are integration with LIMS (Laboratory Information Management System) and metabolite identification. While the problem of data management is recognized as important in the field of metabolomics, a standard or widely accepted LIMS is currently lacking [70]. One of the most promising systems is Sesame, originally developed for structural genomics, whose component LAMP provides LIMS for metabolomic data [71]. Possible future extensions of PyMS include linking with a standard LIMS to achieve integrated data processing and management [70]. Metabolite identification is widely recognized as one of the key challenges in metabolomics [72-74]. A number of standard databases for small molecule identification exists [3], and in recent years MS databases focused on metabolites are emerging [75-78]. We are currently working on PyMS modules for GC-MS spectral matching and metabolite identification, as the priority for future development.

## Conclusions

PyMS is modular software for processing of chromatography-mass spectrometry data developed in Python, an object oriented language widely used in scientific computing. PyMS is a library of functions written in Python, thus seamlessly integrating MS data processing with the capabilities of a general purpose programming language. PyMS currently implements a complete pipeline for the processing of GC-MS data, including reading of standard data formats (ANDI-MS/NetCDF and JCAMP-DX), noise smoothing, baseline correction, peak detection, peak deconvolution, peak integration, and peak alignment by dynamic programming. PyMS is implemented as a nested hierarchy of Python sub-packages, allowing any functionality to be selectively invoked, disregarding any other functionality, i.e., required functions are loaded and then executed within the Python programming environment as needed. PyMS aims to decouple processing methods from interactive processing and visualization, to allow effective scripting and processing in batch mode.

## Availability and requirements

PyMS is released as open source (GNU GPL2 license), and is provided with an extensive User Guide, tutorials, and the library of ready-to-run scripts including the sample data (Table 1). A Web-based documentation system accessible over the Internet provides descriptions of PyMS APIs. The data used in this study, together with suitable metadata, has been deposited into the EMBL-EBI MetaboLights database (http://www.ebi.ac.uk/metabolights/).

**Project name:** PyMS

**Project home page:**http://code.google.com/p/pyms/

**Operating system(s):** Platform independent

**Programming language:** Python

**Other requirements:** NumPy, Netcdf, Pycdf, Pycluster, matplotlib, tcl, tk

**License:** GNU GPL2

**Any restrictions to use by non-academics:** No

## Competing interests

The authors declare that they have no competing interests.

## Authors’ contributions

SOC, AI, QW, LH, MO, TE, and VAL were involved in code development, implementation, and testing. DDS, DLT, AB, UR, and MJM contributed to the design of experiments. DDS prepared the samples, performed GC-MS experiments, and analyzed the data. SOC and DDS performed comparative analyses of different software packages. LH and BA developed MPI parallel capabilities. VAL drafted the manuscript, with contribution from SOC, DDS, AB, and MJM. All authors read and approved the final manuscript.

## Supplementary Material

Additional file 1 ** Table of signals shown in Figure 3. **The table lists signals shown in **Figure 3**. The tables lists signals present in the data as delineated by manual analysis and shown in Figure 3. For each signal (**a**) the retention time and five top m/z ions are given; (**b**) it was marked whether it was found by each of the programs (PyMS, AMDIS, AnalyzerPro, XCMS). Click here for file

Additional file 2 ** Table of signals shown in Figure 4. **The table lists signals shown in **Figure 4**. The tables lists signals present in the data as delineated by manual analysis and shown in Figure 4. For each signal (**a**) the retention time and five top m/z ions are given; (**b**) it was marked whether it was found by each of the programs (PyMS, AMDIS, AnalyzerPro, XCMS). Click here for file

Additional file 3 ** Table of signals shown in Figure 5. **The tables lists signals present in the data as delineated by manual analysis and shown in **Figure 5**. For each signal (**a**) the retention time and five top m/z ions are given; (**b**) it was marked whether it was found by each of the programs (PyMS, AMDIS, AnalyzerPro, XCMS). Click here for file

Additional file 4 ** Table of signals shown in Figure 6. **The table lists signals shown in **Figure 6**. The tables lists signals present in the data as delineated by manual analysis and shown in Figure 6. For each signal (**a**) the retention time and five top m/z ions are given; (**b**) it wasmarked whether it was found by each of the programs (PyMS, AMDIS, AnalyzerPro, XCMS). Click here for file
