# Supplementary material for: PyMS: a Python toolkit for processing of gas chromatography-mass spectrometry (GC-MS) data. Application and comparative study of selected tools
Source: BMC Bioinformatics. 2012 May 30;13:115. doi: 10.1186/1471-2105-13-115 (PMC3533878; doi:10.1186/1471-2105-13-115)
Supplement: Additional file 1 — Table of signals shown in Figure 3. The table lists signals shown in Figure 3. The tables lists signals present in the data as delineated by manual analysis and shown in Figure 3. For each signal (a) the retention time and five top m/z ions are given; (b) it was marked whether it was found by each of the programs (PyMS, AMDIS, AnalyzerPro, XCMS). [file 1471-2105-13-115-S1.pdf]

Rt (mins)	Ion	Intensity	PyMS	AnaylzerPro	XCMS	AMDIS
21.45	350	4661.86	x	√	x	√
	351	1771.4				
	156	1560.53				
	352	980.27				
	133	790.71				
21.61	128	64260.78	√	√	√	√
	243	41408.14				
	115	31721.64				
	141	26947.05				
	100	19575.03				
21.93	218	13448.57	√	√	x	√
	320	7121.77				
	232	5691.06				
	133	2895.09				
	219	2680.86				
22.36	190	21208.45	√	√	x	√
	100	12982.09				
	75	7030.10				
	72	6541.92				
	160	6082.27				
22.45	233	263598.81	√	√	√	√
	245	160759.34				
	133	97438.90				
	148	87032.88				
	190	80647.48				
22.7	232	10991.14	x	√	x	√
	233	2352.47				
	234	1054.62				
	144	1004.97				
	75	586.29				
22.78	191	64775.17	√	√	√	√
	244	15214.69				
	192	11171.79				
	75	6422.36				
	69	6129.25				
22.80	57	56327.90	√	√	√	√
	71	38976.70				
	243	34677.19				
	85	28391.47				
	115	26967.10				
23.04	188	11543.2	x	√	x	√
	84	2661.09				

	189	1594.77				
	156	853.75				
	56	705.75				
23.18	176	1163459.27	√	√	√	√
	128	384991.88				
	177	160403.23				
	61	140989.85				
	293	127564.96				
23.21	232	184533.19	√	√	√	√
	233	36636.88				
	234	15992.52				
	306	5280.20				
	216	5069.71				
23.26	156	2267023.76	√	√	√	√
	157	292266.81				
	258	274042.30				
	230	217081.29				
	158	95046.98				
23.47	175	13292.76	√	√	√	√
	130	4979.94				
	191	4035.77				
	120	2903.59				
	320	2769.25				
23.55	174	9734.02	√	√	√	√
	84	8529.55				
	75	4665.77				
	56	3075.05				
	158	2953.82				
23.60	142	4519.30	√	√	x	√
	75	3638.78				
	140	3472.98				
	156	2186.35				
	89	1194.65				
23.88	120	44570.32	√	√	√	√
	146	19123.96				
	91	5884.69				
	130	5811.12				
	75	5698.08				
24.00	75	5706.74	√	√	x	√
	198	4483.51				
	89	3415.38				
	156	2272.97				

	55	2138.46				
24.30	218	10082.16	√	√	x	√
	261	7620.26				
	162	2822.54				
	219	2144.05				
	100	1994.51				
24.62	198	125498.88	√	√	√	√
	75	116807.89				
	304	65591.14				
	156	64402.91				
	288	60730.96				
24.70	186	32996.21	√	√	x	√
	142	32560.01				
	288	6929.52				
	216	6138.08				
	187	5128.53				
24.80	218	13287.95	√	√	x	√
	261	9467.17				
	219	2806.87				
	162	2805.33				
	100	2567.84				
