# Supplementary material for: PyMS: a Python toolkit for processing of gas chromatography-mass spectrometry (GC-MS) data. Application and comparative study of selected tools
Source: BMC Bioinformatics. 2012 May 30;13:115. doi: 10.1186/1471-2105-13-115 (PMC3533878; doi:10.1186/1471-2105-13-115)
Supplement: Additional file 2 — Table of signals shown in Figure 4. The table lists signals shown in Figure 4. The tables lists signals present in the data as delineated by manual analysis and shown in Figure 4. For each signal (a) the retention time and five top m/z ions are given; (b) it was marked whether it was found by each of the programs (PyMS, AMDIS, AnalyzerPro, XCMS). [file 1471-2105-13-115-S2.pdf]

Rt (mins)	Ion	Intensity	PyMS	AnaylzerPro	XCMS	AMDIS
30.89	103	111855.79	√	√	√	√
	307	109464.19				
	217	103437.07				
	308	30600.90				
	277	20868.96				
31.10	307	79983.77	√	√	√	√
	217	77564.75				
	103	70295.11				
	308	21891.68				
	218	15262.36				
31.17	319	242223.09	√	√	√	√
	205	132534.55				
	320	71073.81				
	160	70114.01				
	217	50839.83				
31.25	319	203946.76	√	√	√	√
	205	126294.73				
	217	66616.00				
	320	60912.37				
	160	47295.05				
31.38	319	232798.69	√	√	√	√
	205	127817.78				
	160	71103.18				
	320	68891.81				
	217	52384.84				
31.44	319	36708.46	√	√	√	√
	205	21273.29				
	160	11733.90				
	320	10880.66				
	217	7890.43				
31.66	57	85103.46	√	√	√	√
	71	64981.99				
	85	47703.31				
	55	18962.16				
	154	15662.88				
31.68	319	56065.46	√	√	√	√
	205	34887.73				
	320	16766.18				
	217	16612.55				
	154	15946.39				

31.75	319	41892.18	√	√	√	√
	205	24186.07				
	320	12750.63				
	160	11429.43				
	217	8952.50				
31.90	154	141424.05	√	√	√	√
	254	57973.62				
	155	26179.94				
	255	13359.99				
	356	10067.70				
31.93	174	110589.29	√	√	√	√
	317	94875.14				
	156	35432.75				
	318	30298.18				
	230	29908.03				
31.98	319	352055.99	√	√	√	√
	205	177717.62				
	217	132698.65				
	320	104326.66				
	103	65458.34				

