# Supplementary material for: PyMS: a Python toolkit for processing of gas chromatography-mass spectrometry (GC-MS) data. Application and comparative study of selected tools
Source: BMC Bioinformatics. 2012 May 30;13:115. doi: 10.1186/1471-2105-13-115 (PMC3533878; doi:10.1186/1471-2105-13-115)
Supplement: Additional file 3 — Table of signals shown in Figure 5. The tables lists signals present in the data as delineated by manual analysis and shown in Figure 5. For each signal (a) the retention time and five top m/z ions are given; (b) it was marked whether it was found by each of the programs (PyMS, AMDIS, AnalyzerPro, XCMS). [file 1471-2105-13-115-S3.pdf]

Rt (mins)	Ion	Intensity	PyMS	AnalyzerPro	XCMS	AMDIS
8.71	61	61919.35	x	√	√	√
	104	52226.56				
	56	51717.97				
	75	44499.92				
	160	36035.31				
8.73	104	704786.15	√	√	√	√
	61	652518.31				
	56	607251.74				
	75	486661.62				
	160	476052.43				
8.74	174	53796.98	√	√	√	√
	248	30973.28				
	175	11053.26				
	290	9941.1				
	249	7989.07				
8.78	174	35214.84	√	√	√	√
	175	7006.43				
	155	6255.12				
	59	5760.92				
	74	5686.73				
8.84	257	117660.37	√	√	√	√
	258	27568.21				
	75	21511.17				
	104	16437.26				
	61	15831.09				
8.86	243	130540.07	√	√	√	√
	244	28242.18				
	75	21245.7				
	61	15175.56				
	245	13880.63				
8.91	174	77874.43	√	x	x	√
	86	21749.92				
	175	14797.24				
	156	14563.11				
	160	13941.55				
8.92	59	19734.46	√	x	√	√
	243	19131.54				
	154	16664.27				
	104	15807.57				
	130	15242.44				
8.93	218	22949.66	√	√	√	√
	75	15126.82				
	160	14138.53				
	320	7807.01				
	133	7059.04				
8.98	158	72764.45	√	√	√	√

	68	67279.34				
	155	16581.67				
	160	16184.26				
	159	12013.33				
8.99	255	158730.37	√	x	√	√
	256	36418.23				
	257	18443.18				
	104	16620.2				
	270	16411.92				
9	233	126537.91	√	√	√	√
	75	107703.89				
	74	83087.07				
	133	80671.47				
	245	73220.77				
9.02	104	16621.2	√	√	x	√
	217	15465.32				
	103	14281.03				
	117	10349.16				
	205	10129.5				
