# Supplementary material for: PyMS: a Python toolkit for processing of gas chromatography-mass spectrometry (GC-MS) data. Application and comparative study of selected tools
Source: BMC Bioinformatics. 2012 May 30;13:115. doi: 10.1186/1471-2105-13-115 (PMC3533878; doi:10.1186/1471-2105-13-115)
Supplement: Additional file 4 — Table of signals shown in Figure 6. The table lists signals shown in Figure 6. The tables lists signals present in the data as delineated by manual analysis and shown in Figure 6. For each signal (a) the retention time and five top m/z ions are given; (b) it wasmarked whether it was found by each of the programs (PyMS, AMDIS, AnalyzerPro, XCMS). [file 1471-2105-13-115-S4.pdf]

Rt (mins)	Ion	Intensity	PyMS	AnalyzerPro	XCMS	AMDIS
7.83	116	71888.43	√	√	√	√
	132	68591.7				
	57	16969.41				
	144	11934.9				
	80	8507.89				
7.85	205	1102339.37	√	√	√	√
	117	636852.17				
	103	528148.3				
	133	397423.45				
	218	297962.75				
7.88	158	4058907.25	√	√	√	√
	159	600902.34				
	232	197144.35				
	74	192228.67				
	75	190012.83				
7.89	299	3878453.65	√	√	√	√
	300	970830.45				
	314	615285.01				
	301	526580.12				
	133	364493.21				
8.01	158	531088.16	√	√	√	√
	218	96658.41				
	159	77247.38				
	75	35251.57				
	74	33932.75				
8.03	117	22160.51	√	√	√	√
	130	14139.97				
	75	13733.86				
	219	9624.78				
	57	8559.53				
8.08	142	671743.31	√	√	√	√
	143	90111.19				
	75	28338.14				
	216	27851.79				
	144	27657.7				
8.1	174	4680064.34	√	√	√	√
	248	935970.4				
	175	889935				
	86	523270.65				
	133	399538.63				
8.12	75	556350.51	√	√	√	√
	148	422788.91				
	247	279113.05				
	149	231568.62				
	55	197423.76				
8.17	189	57236.28	√	√	√	√

	205	37784.72				
	292	37292.38				
	133	34525.84				
	103	34376.56				
